# Dengue in the United States of America: A Worsening Scenario?

**DOI:** 10.1155/2013/678645

**Published:** 2013-06-20

**Authors:** Germán Añez, Maria Rios

**Affiliations:** Laboratory of Emerging Pathogens, DETTD/OBRR/CBER, U.S. Food and Drug Administration, Bethesda, MD 20892, USA

## Abstract

Dengue is a febrile illness caused by any of the four dengue virus types (DENV-1 to -4, genus *Flavivirus*, family Flaviviridae) mainly transmitted by the mosquito *Aedes aegypti*. DENV can be transmitted by blood transfusion. Dengue has been historically present in the continental United States (US), in the state of Hawaii, and in the US insular territories in the Caribbean and the Pacific. During the second half of the 20th century, most of the cases reported in the US were imported cases brought to the country by travelers. Since 2009, cases of autochthonous dengue have been recognized in the state of Florida after 75 years of absence, followed by intensification of transmission in endemic places including the US territories of US Virgin Islands and Puerto Rico, which experienced a large dengue epidemic in 2010. The widespread distribution of dengue mosquito vectors, deficient mosquito control measures and increased frequency of DENV-infected visitors to the US coming from dengue-endemic locations or places experiencing epidemics appear to be jointly responsible for the emergence and reemergence of dengue in the US and its territories.

## 1. Introduction

Dengue, the most prevalent arthropod-borne viral disease in the world, is an acute, febrile disease caused by any of the four dengue virus types (DENV-1 to -4, genus *Flavivirus*, family Flaviviridae) [1]. DENV is naturally transmitted by mosquitoes from the genus *Aedes,* mainly by the urban species *Aedes aegypti, *and in some geographical regions by* Aedes albopictus*. Infection by dengue viruses can be asymptomatic or cause disease of variable degree of severity. Dengue ranges from a mild, influenza-like illness known as dengue fever (DF) to a severe and potentially life-threatening condition called dengue hemorrhagic fever (DHF). DHF can ultimately evolve to hypovolemic shock (dengue shock syndrome (DSS)) and death [2]. DHF and DSS are classified as severe dengue in the newest World Health Organization (WHO) dengue clinical classification [3]. 

DENV can also be transmitted by transfusion of blood and blood components and by solid organ transplants containing infectious virus [4–6]. Dengue is endemic in most countries of tropical America, the Caribbean and Southeast Asia, and causes episodic epidemics in islands of the Pacific and in Africa [7, 8]. Many DENV endemic regions are hyperendemic, which is defined by the circulation of all DENV types in a specific geographical area. This may increase the opportunity for occurrence of secondary infections with increased clinical severity via antibody-dependent enhancement of the infection by sub-neutralizing concentrations of anti-dengue antibodies in individuals previously infected with a different dengue type (heterologous infection) [9].

Approximately, 50–100 million cases of dengue were calculated to occur each year around the globe [3]. However, the newest estimates raise these numbers to about 390 million cases per year (range 284–528), from which approximately 96 (range 67–136) will present clinical manifestations of any severity [10]. Despite its low lethality rates, dengue causes severe economic and social disruption and has profound impact on the welfare of regions affected by the disease since it is endemic mostly in developing countries where healthcare systems have limited resources [11–13]. After many years of continuous efforts, there are still neither effective vaccines nor specific antiviral treatments available against DENV [14]. 

The mosquitoes that serve as DENV vectors have been able to reach sub-tropical and temperate regions, including North America and countries in Europe [15–17]. In addition, DENV brought in by infected travelers has been able to establish autochthonous infection cycles in some of these countries. DENV is considered the most common cause of febrile illness in travelers returning to the USA from destinations in the Americas and Asia [18].

The scope of this review is to provide an analysis of the epidemiology of dengue in the United States and its territories, with emphasis on the changes in dengue activity in the last decade and on aspects on the molecular epidemiology of currently circulating DENV. 

## 2. Dengue in the United States

Dengue is thought to have been present in the USA since the end of the 18th century, when Dr. Benjamin Rush, a physician and signatory of the US Declaration of Independence, described a disease resembling dengue fever in Philadelphia during 1780 [27]. During the first half of the 20th century, a number of dengue outbreaks were reported in the continental USA, especially in the gulf and southeastern states (i.e., Alabama, Florida, Georgia, Louisiana, Mississippi and Texas), in the state of Hawaii, and in the USA Territories in the Caribbean and the Pacific Ocean (Table 1).

The continental USA comprises the 48 contiguous states and the District of Columbia, and the non-contiguous states of Alaska in North America and Hawaii in the Pacific Ocean and a number of unincorporated territories including Puerto Rico and the US Virgin Islands (USVI) in the Caribbean and American Samoa, Guam, and the Northern Mariana Islands in the Pacific Ocean [19].

**Figure 1:**
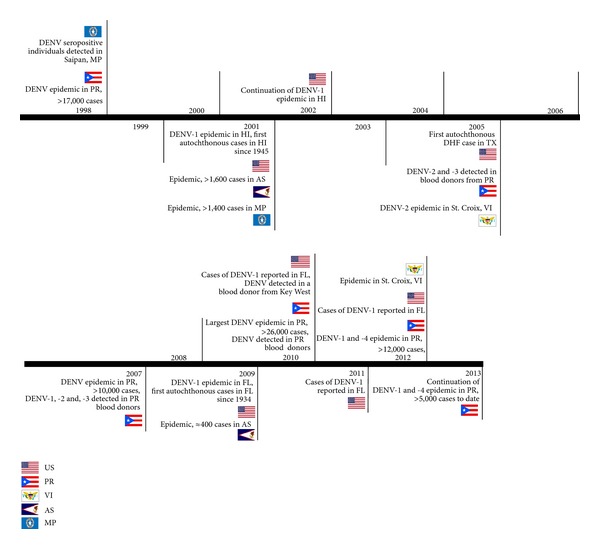
Timeline of selected recent dengue activity in the U.S. and its territories, 1998–2013. U.S.: United States; representing the states of Florida (FL), Hawaii (HI) and Texas (TX), P.R.: Puerto Rico, V.I.: U.S. Virgin Islands, A.S.: American Samoa, M.P.: Northern Mariana Islands. Numbers shown represent dengue reported cases.

Autochthonous dengue has been reported sporadically in the Mexico-Texas border, where indigenous cases of dengue reappeared in 1980 after more than 30 years of absence. Since then, most dengue cases reported in the USA have been imported, brought by infected travelers returning from visits to endemic countries or places experiencing dengue epidemics. Dengue has also been present in the state of Hawaii, the US territories in the Pacific (American Samoa, Guam, and Northern Mariana Islands) and the territories of Puerto Rico and USVI. In 2009, after over 70 years of absence, autochthonous dengue reappeared in the state of Florida (Figure 1). 

**Figure 2:**
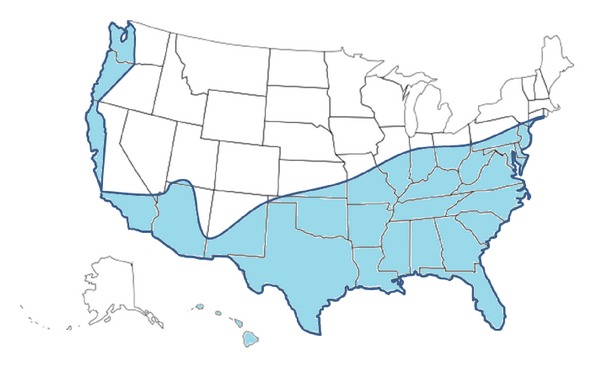
Map of the U.S. showing the areas at risk of dengue outbreaks, based on the approximate distribution of dengue mosquito vectors *Aedes aegypti *and *Aedes albopictus*. Map adapted from [28, 29]. The delimited area represents the approximate geographical area in which either dengue mosquito vector (*Aedes aegypti *and/or *Aedes albopictus*) have been found present in the USA and are therefore considered to be at risk for the establishment of dengue outbreaks. The noncontiguous states of Alaska and Hawaii are not shown at scale. U.S. territories are not shown.

In all those instances, dengue outbreaks were facilitated by the presence of the mosquito vector, the favorable climatic conditions for its subsistence, and the presence of susceptible individuals. The dengue mosquito vectors *Aedes aegypti *and *Aedes albopictus* have been reported to be present in several counties in the USA, especially in the Southern and Southeastern regions of the country [28] (Figure 2).

### 2.1. Dengue in the Mexico-Texas Border

The first autochthonous case of dengue reported in the USA since 1945 occurred in Brownsville, Texas in 1980 when DENV-1 was isolated from a 5-year-old girl that did not have history of travel outside Brownsville (Table 1). Brownsville is located across the Rio Grande from the city of Matamoros in the state of Tamaulipas, Mexico where many years of dengue activity had been observed [21]. 

Subsequently, surveillance studies reported 63 additional dengue cases (all caused by DENV-1) that were laboratory confirmed in Texas, 52 of which occurred in counties contiguous to the Mexico-Texas border [30]. Six years later, autochthonous transmission of DENV-1 was again reported in at least 9 individuals in Texas [22]. The Mexico-Texas border region is at risk for dengue endemicity due to the presence of the competent mosquito vectors *Aedes aegypti *and *Aedes albopictus* and to the circulation of all DENV types (DENV-1 to -4) in recent years [31]. In sum, dengue has been detected in residents of seven Texas counties: Bee, Cameron (where Brownsville is located), Hidalgo, Maverick, Nueces, Travis, and Webb [23, 30, 32]. 

In 2005, a woman who had not traveled to Mexico in the months before the onset of dengue symptoms developed DHF, and this was the first case of autochthonous DHF reported in Texas. During that year, a large dengue epidemic developed in the neighboring Mexican state of Tamaulipas with more than 1,200 dengue cases of which 223 (18%) were classified as DHF. Meanwhile, 25 dengue cases were reported in Brownsville, of which at least three were locally acquired [32]. The disparities in the number of cases observed on different sides of the border have been explained by the higher standard of living in the USA including the use of air-conditioners and the presence of nets in doors and windows, which remain closed most of the time reducing the density of indoor mosquitoes thus minimizing the risk of mosquito bites [33].

### 2.2. Dengue in Florida

After 75 years without reports of dengue activity in Florida, a case was identified in 2009 in a New York patient who had traveled to Key West (Table 1). This individual had not traveled to any other place either in the USA or abroad before the onset of symptoms, and the case was laboratory-confirmed by the Centers for Disease Control and Prevention (CDC) using RT-PCR. A serosurvey conducted in 2009 revealed that at least 5.4% of the residents of Key West had serological evidence of recent dengue infection [24]. 

According to the Florida State Department of Health, between 2009 and 2012, a total of 103 autochthonous dengue cases have been reported in Florida including those from the outbreak of 2009. Of these, 27 were reported in 2009 in Key West (Monroe county), 65 during 2010 (Broward, Miami-Dade, and Monroe counties), 7 in 2011 (Hillsborough, Martin, Miami-Dade, and Palm Beach counties) and 4 in 2012 (Miami-Dade, Seminole, and Osceola counties) [25, 26], (Table 2). 

**Figure 3:**
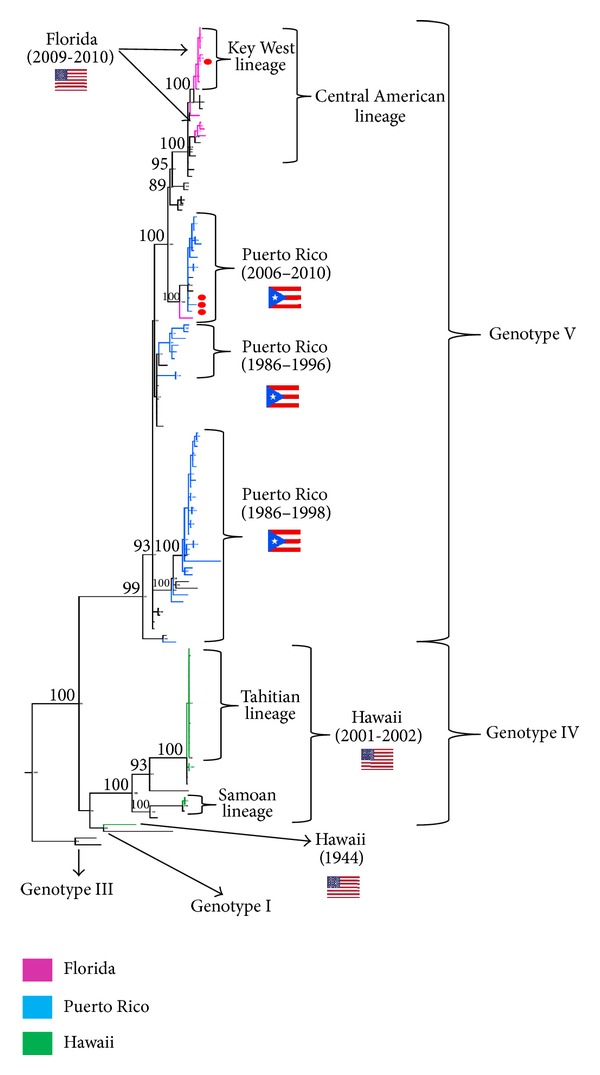
Phylogeny of DENV-1 in the USA and Puerto Rico. A consensus phylogenetic tree (50% majority rule) was obtained by Bayesian phylogenetic analysis (Mr. Bayes, v. 3.2.) based on the envelope protein gene. Analysis included sequences of strains from Hawaii (2001-2002) (*n* = 21), Florida (2009-2010) (*n* = 15), and Puerto Rico (1986–2010) (*n* = 45) available in the GenBank database and representative sequences from DENV-1 genotypes I, III–V (*n* = 44). DENV-2, DENV-3, and DENV-4 were used to root the tree (not shown). Bayesian posterior probability values (>80) are shown for the principal nodes. Taxa are highlighted according to its geographical origin: Hawaii (green), Florida (pink), and Puerto Rico (light blue). A red dot identifies sequences obtained from blood donors.

DENV-1 was detected in mosquito pools collected in Key West [49] and in a blood donor from Key West during 2010 [50]. The strains isolated from these two specimens were sequenced, and subsequently, a number of DENV-1 strains obtained from samples from dengue cases from Key West/Monroe county (8 patients) and one strain each from patients from Broward, Miami-Dade, Orange, and Pinellas counties were sequenced [51]. All DENV-1 strains from Florida from 2009-2010 sequenced to date (obtained from mosquito pools, dengue cases, and a blood donor) belong to the genotype V of the virus and have phylogenetic relationships with Central American strains; however, all but one of the strains sequenced from Key West grouped together in the phylogenetic trees, suggesting that *in situ* microevolution has occurred and that this Key West sublineage was transmitted during 2009 and 2010 (Figure 3) [50, 51]. Sequences of DENV-1 strains obtained from cases acquired in other counties in Florida clustered in a separate phylogenetic group, more closely related to Central American strains (i.e., from Costa Rica, Mexico, and Nicaragua). Only one DENV-1 strain from Key West was found clustering outside the Key West lineage, which suggests that introduction of at least two different DENV-1 lineages occurred in Key West during 2009-2010 epidemics [51] (Figure 3). 

The dengue outbreak of 2009-2010 has been linked to *Aedes aegypti*, which is prevalent in Monroe county where Key West is located. Although *Aedes albopictus* is present in Florida, it has not become established in Monroe county. Mosquitoes from both *Aedes *species obtained from colonies in the state of Florida were experimentally infected with a DENV-1 strain isolated from Key West, and vector competence for this virus was assessed. There were no significant differences in the rates of infection, dissemination, and transmission between these two mosquito species occurring in Florida. The entire genome of the DENV-1 specimen utilized in this vector competence study has been sequenced [16] and shown to cluster within the Key West lineage (Figure 3).

In addition to the autochthonous cases, an important number of dengue imported cases (392) from travelers to countries in Central and South America, the Caribbean, Africa, the Middle East, and South and Southeast Asia have been reported in Florida since 2009 [25, 26] (Table 2). The wide geographical area of origin of dengue imported cases in Florida reflects the importance of the Miami International Airport as a gateway to the USA and a possible route of entry for these and other pathogens.

### 2.3. Dengue in Hawaii

The first large dengue epidemic on record occurred in Hawaii in the mid-19th century. Another large epidemic occurred in 1903 with approximately 30,000 cases reported [34, 35]. *Aedes aegypti *was the vector implicated in those dengue outbreaks. But at a certain point during the early 20th century, *Aedes albopictus *was introduced in these islands and displaced *Aedes aegypti* [52].

A DENV-1 epidemic occurred in Hawaii from 1943 to 1944, with almost 1,500 cases reported [36, 53]. As in the continental USA no autochthonous cases of dengue were reported after 1945 and imported dengue cases were reported at a low frequency in Hawaii [54]. In 1995, two German travelers developed symptoms compatible with dengue infection after a trip to Hawaii [46] (Table 3). 

However, in 2001, following 56 years without reports of autochthonous dengue cases, an epidemic occurred in the island of Maui. A total of 122 laboratory-confirmed dengue cases were reported between 2001 and 2002, 92 of which occurred in Maui, 26 in Oahu, and 4 in Kauai. DENV-1 was the type identified in viral isolates obtained from 15 cases from Hawaii, and the mosquito vector implicated in this epidemic was *Aedes albopictus* [54]. 

Molecular epidemiologic studies suggest that at least two distinct DENV-1 strains were introduced in Hawaii during the 2001-2002 epidemics, although most of the isolates analyzed belong to the “Pacific subtype” (genotype IV) of DENV-1, and cluster together with Tahitian DENV-1 strains, suggesting that Tahiti was the source of these strains. In contrast, the same study reported a single DENV-1 isolate from Hawaii obtained during 2001 from a traveler to Samoa that clustered outside the Tahitian cluster and that closely associated with a DENV-1 isolate previously obtained from another traveler to Samoa [39] (Figure 3).

### 2.4. Dengue in American Samoa, Guam and Northern Mariana Islands

Dengue was reported in American Samoa and Guam before 1950 [38] (Table 3). The main vector, *Aedes aegypti,* had been considered as eradicated from Guam since 1944. However, *Aedes albopictus* is abundantly present in the island [37]. Guam has not had dengue outbreaks in recent times, but it is at risk of dengue epidemics due to the presence of *Aedes albopictus *mosquitoes and the arrival of infected travelers from neighboring islands experiencing dengue activity.

Conversely, American Samoa had dengue epidemics in 1972 (DENV-2), 1975 (DENV-1), and 1995-1996 (DENV-3) [38, 40, 71, 72], while some dengue cases were reported in 1997 [41]. In 2001, more than 1,600 dengue suspected cases caused by DENV-1 were reported [42, 47], with 237 hospitalizations and three deaths due to DHF. In 2007, at least 63 dengue cases were confirmed, 23 of which were hospitalized [47]. Dengue activity was reported in 2008-2009; in 2009, an important outbreak of about 400 confirmed cases (for an incidence of 644/100,000 inhabitants) was registered [48]. 

In 2010, less than 100 dengue cases were reported for an incidence of 77/100,000 inhabitants [8, 48]. Epidemiological surveillance performed in 794 serum samples collected in three islands of American Samoa in 2010 revealed that 759 (95.6%) of the tested individuals were positive for IgG antibodies to dengue [44]. The dengue vectors, *Aedes aegypti *and* Aedes albopictus*, are present in American Samoa [73].

There are reports indicating that individuals from the Northern Mariana Islands, without travel history, were seropositive for DENV (IgM and IgG) in 1998 in the island of Saipan [43], where DENV-2 was the DENV type implicated in those infections [42]. Furthermore, these islands suffered a dengue outbreak in 2001 with more than 1,400 cases reported [45]. Since then, no other dengue cases have been reported.

### 2.5. Dengue in US Virgin Islands

**Figure 4:**
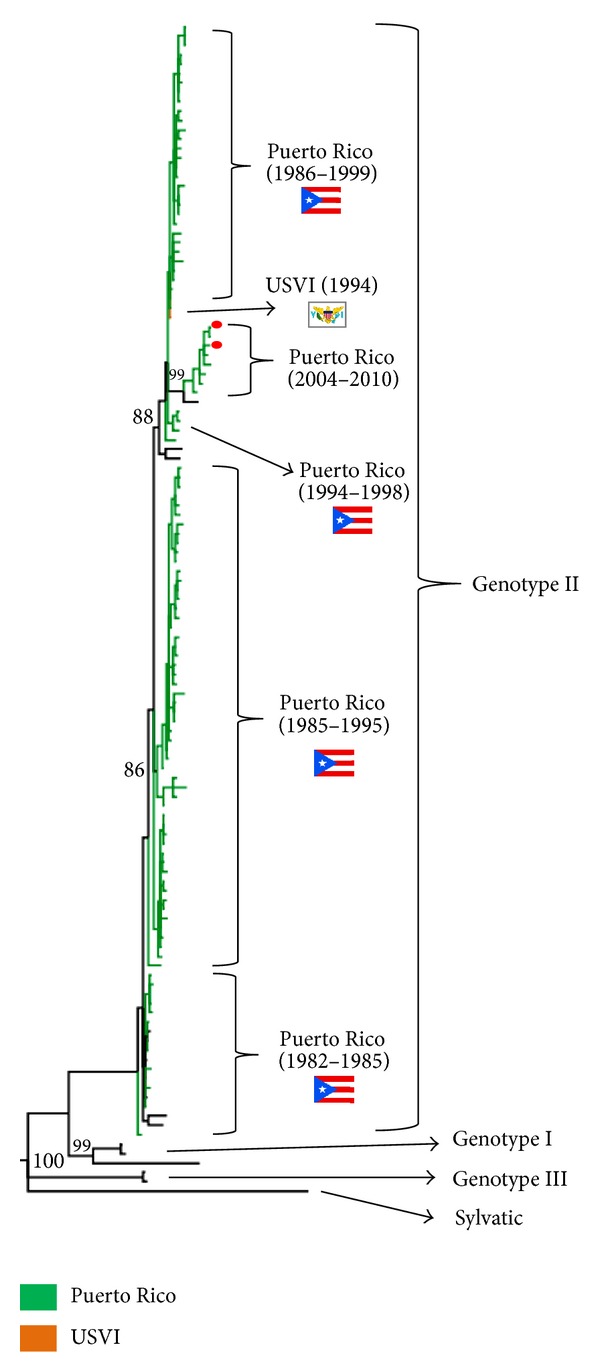
Phylogeny of DENV-4 in Puerto Rico and the US Virgin Islands (USVI). A consensus phylogenetic tree (50% majority-rule) was obtained by Bayesian phylogenetic analysis (Mr. Bayes, v. 3.2.) based on the envelope protein gene. Analysis included sequences of strains from USVI (1994) (*n* = 1) and Puerto Rico (1982–2010) (*n* = 115) available in the GenBank database, and representative sequences from DENV-4 genotypes I–III and Sylvatic (*n* = 12). DENV-1, DENV-2, and DENV-3 were used to root the tree (not shown). Bayesian posterior probability values (>80) are shown for the principal nodes. Taxa are highlighted according to its geographical origin: Puerto Rico (green) and USVI (orange). A red dot identifies sequences obtained from blood donors.

Dengue has been endemic in the USVI since at least 1924, when the first documented dengue epidemic in the Caribbean is thought to have started in the USVI [20]. These islands are located in close proximity to Puerto Rico, and include the four inhabited islands of St. Croix, St. Thomas, St. John and Water Island. Dengue outbreaks occurred in 1978 (DENV-1) and in 1990, this time involving DENV-1, DENV-2 and DENV-4; 1990 was the year of the first occurrence of DENV-4 in the USVI [20]. The sequence of a DENV-4 strain isolated in 1994 from St. Croix closely associated with strains that were circulating in Puerto Rico around the same time (Figure 4).

Forty dengue cases were reported in the island of St. Thomas in 2004. In 2005, a DENV-2 epidemic was reported in St. Croix, with 331 suspected cases of which 37% were laboratory-confirmed [65]. The sequence of a DENV-2 strain from the 2005 epidemic clustered within the clade IA of the American/Asian genotype, together with a number of strains from the Caribbean and South America [74].

During November 2012, 27 dengue cases were reported in the island of St. Croix, some of which were later laboratory confirmed. A serosurvey found that around 20% of the students and staff from a school in the island were positive for IgM antibodies to dengue; four students were positive for DENV-1 or DENV-4 RNA by PCR [69]. There is no available genetic information about the DENV strains circulating in St. Croix in 2012. 

### 2.6. Dengue in Puerto Rico

Dengue is endemic in Puerto Rico, and cases have been reported every year for more than 50 years (Table 4). The public health definition of a dengue epidemic in Puerto Rico is based on a historical average: the event is called an epidemic when the number of cases detected by the passive surveillance system is higher than the 75th percentile of the distribution of cases for the same epidemiological week in previous years [75]. 

A large epidemic of DENV-3 involving approximately 27,000 individuals was reported in 1963-1964 [55, 56]. Another epidemic caused by DENV-2 occurred in 1969 [57]. Subsequently, sporadic dengue cases caused by DENV-2 were reported during the early 1970s [57]. In 1975, a case of DHF (associated with DENV-2) was reported for the first time in Puerto Rico and in the Western Hemisphere [20]. The outbreak in 1977 was caused by DENV-2, DENV-3 and DENV-1 (DENV types appear in order of frequency of detection), and that was the first documented occurrence of DENV-1 in the island [58]. 

The following year (1978), another dengue outbreak was reported, this time caused primarily by DENV-1 [59]. In 1981, an outbreak caused by DENV-4, and DENV-1 (in order of frequency) was reported and that was the first report of DENV-4 in the Americas [60]. Additional outbreaks followed in 1982 (DENV-4) [60, 61] and 1985-1986 (DENV-4, DENV-1 and DENV-2, in order of frequency of detection), when a dengue epidemic caused 31 DHF cases and 3 deaths [62] (Table 4). 

In 1994-1995, approximately 24,000 cases of dengue were reported in Puerto Rico, with circulation of DENV-2, DENV-4 and DENV-1 (in order of frequency of detection) [63]. In 1998, over 17,000 dengue cases were reported, and for the first time the co-circulation of all four DENV types was observed. During this outbreak, DENV-3 was detected in the island after an absence of 20 years [64] (Table 4). 

In 1999, all four DENV types co-circulated in the island resulting in 4,993 reported cases. The 2000 epidemic had co-circulation of DENV-1, DENV-2, and DENV-3 and resulted in 2,433 dengue cases reported [20]. DENV-3 was the predominant type in the inter-epidemic period between 1999 and 2003, to be later displaced by DENV-2 as the predominantly DENV type detected during the inter-epidemic period spanning from 2004 to 2006 [66].

In 2007, a large epidemic (10,508 suspected dengue cases, with 227 fulfilling criteria for DHF) occurred after an interepidemic period of almost ten years; this epidemic was the second outbreak to have co-circulation of all DENV types, after the 1998 epidemic. During the 2007 dengue epidemic, 2,175 individuals tested positive for DENV RNA, of which 62% was infected with DENV-3, 31% with DENV-2, 6% with DENV-1, and 1% with DENV-4. DENV-1, and DENV-4 reappeared after approximately 9 years of absence (Table 4). Overall, an increased incidence of severe disease was reported in the 2007 epidemic when compared with previous outbreaks, resulting either from more efficient reporting of severe cases or from a true increase in the incidence of severe dengue [66].

The largest epidemic of dengue in Puerto Rico's history occurred during 2010, with almost 27,000 suspected dengue cases, of which more than 12,000 individuals (about 47% of tested cases) were laboratory-positive; more than 1,300 cases were classified as severe dengue, and there were 40 dengue-associated deaths (Table 4). The DENV types implicated in this epidemic were in order of frequency DENV-1 (69%), DENV-4 (23.7%), and DENV-2 (7.3%), with only two DENV-3 cases reported (<0.1%). The 2010 epidemic is considered the longest-lasting dengue outbreak ever registered in Puerto Rico, with cases starting to appear during the first week of 2010, peaking around August and returning to levels below the historical average in December of that year [67].

In 2012, an epidemic resulted in 12,877 suspected cases reported, of which 5,652 (44%) were laboratory confirmed. DENV-1 and DENV-4 were the predominant types detected, similar to the 2010 epidemic [68], (Figure 1). This outbreak has continued into 2013, although with a declining trend. Up to April 22, 2013, 5,251 dengue suspected cases had been reported, 2,573 (49%) of which were laboratory-confirmed, and similar to the observed during the 2012 epidemic, DENV-1 (78%) and DENV-4 (21%) were the DENV types predominantly reported [70], (Figure 1).

#### 2.6.1. Epidemiological Data Obtained from Asymptomatic Infections in Blood Donors

Because DENV can be transmitted by blood transfusion [4, 6] and infection can be asymptomatic [1–3], there is great concern about DENV activity among blood donors in endemic places in the absence of overt epidemic and during epidemic times. Prevalence studies in blood donors have been performed in various areas around the world where DENV is endemic [76]. 

A study performed in asymptomatic blood donors from Puerto Rico during the inter-epidemic year 2005 reported that 0.7% (12 out of 16,521) blood donations tested positive for DENV RNA. Of these, three donors were identified by TaqMan as infected with DENV-2 and one with DENV-3, which were the DENV types circulating in the island during that year (Figure 1). Two of the DENV-2 and the DENV-3 strains were isolated in cell culture or by mosquito inoculation [4]. Another study conducted among blood donors during the epidemic year of 2007 found that 29 out of 15,350 donations tested positive for DENV RNA using an assay that detects all four DENV types but does not discriminate between DENV types. Discriminatory real-time PCR detected 12 positive samples: one was identified as DENV-1, four as DENV-2, and seven as DENV-3 (Figure 1). All these 12 samples were infectious in C6/36 cell cultures [6]. 

Study of DENV strains circulating in 2010 performed in blood donors [50] and in symptomatic cases [67] showed that these DENV-1 strains belong to the genotype V (the only one found to circulate in the Americas to date) but were from a lineage different from those that circulated in Puerto Rico during and before 1998 (Figure 3). Likewise, the sequenced DENV-4 strains from 2010 were found to belong to the genotype II (also the only one circulating in the Americas) and are distinct from those that circulated in Puerto Rico in the 1990s, although with less genetic variation than that observed in the newest DENV-1 strains circulating in the island (Figure 4). The results suggest that overall, a clade replacement for DENV-1 and DENV-4 may have occurred at some point in Puerto Rico during the inter-epidemic period between 1999 and 2006 [50, 67]. 

## 3. Conclusions

Dengue has emerged and re-emerged in many locations around the world, including countries in Europe (e.g., France and Croatia) [77, 78] and in North America [24] during the last two decades. Dengue has reemerged and caused epidemics in the continental USA for the first time after several decades of absence, and a worrisome panorama is expected if the trend of transmission continues. Several articles expressing these concerns have been published before and after the re-emergence of dengue in Florida in 2009 [79, 80]. Such concern is augmented by evolving climatic and ecological conditions that favor vector sustainability and by high travel activity with subsequent importation of cases. In fact, cases of dengue in returning travelers to the USA have been on the rise [81], and cases of dengue in countries in Central America, South America, and the Caribbean, very popular destinations for American tourists, have also been on the rise in the past decades [82].

Although the primary mosquito vector for dengue is *Aedes aegypti,* a highly domesticated urban mosquito, the virus can be also transmitted by *Aedes albopictus, *albeit in a less efficient manner. *Aedes albopictus* is probably responsible for the maintenance of dengue in rural/sylvatic cycles in endemic countries [83], and future research should address the biological and epidemiological implications of the displacement and replacement of *Aedes *species in the context of dengue epidemics. 

In the case of the USA, both dengue vector species are widely distributed in the southern parts of the country (Figure 2), and one or another dengue vector is present in all US insular territories [28, 29]. Travel, especially by air, has been considered an important risk factor for the rapid dissemination of pathogens and their vectors in an efficient and rapid manner [84–87]. New promising vector control strategies based on the release of Wolbachia-infected *Aedes aegypti* mosquitoes have been tested in regions of Australia with potential for the occurrence of dengue epidemics and if deemed suitable, this approach could be utilized in endemic regions from Asia and the Americas [88].

Many large cities in the US are important hubs for air travel and therefore receive a high number of individuals potentially infected with pathogens that cause asymptomatic disease, including several arboviruses (e.g., dengue viruses, Japanese Encephalitis virus and Chikungunya virus). Thus, there is an increasing risk of introduction of these “exotic” pathogens to urban conglomerates where mosquito vectors are present or have the potential to become established (e.g., Miami, Atlanta, Baltimore/Washington, D.C., and New York City in the East Coast, and Los Angeles and San Francisco in the West Coast). 

Despite the current economic and budgetary constraints, strict mosquito control policies and activities, that may include both traditional and biological vector control strategies, must be implemented and maintained in localities that have the potential to become the port of entry for these viruses and become the focus for another dengue epidemic in the USA.

## Figures and Tables

**Table 1:** Dengue activity in the continental USA between 1780 and 2013.

Year(s)	Activity reported	References
1780	Dengue suspected in Philadelphia, PA	[19]
1826	USA ports report 1st dengue outbreak	[20]
1827-1828	Epidemic in Southern USA	[20]
1845	Dengue reported in St. Louis, MO	[20]
1850-1851	1st report of dengue epidemic inland (including GA and MS), epidemic in Southern USA, New Orleans, LA, and along South Coast	[20]
1870–1872	Epidemic in Southern USA	[20]
1873	Dengue reported in LA, AL, and MS; *≈*40,000 cases reported in New Orleans	[20]
1879-1880	Epidemic in Southern USA	[20]
1885-1886	Dengue in gulf ports of TX, dengue in Austin, 16,000 estimated cases of 22,000 inhabitants	[20]
1897–1903	Epidemic in Southern USA, TX most heavily affected	[20]
1904	Dengue reported in FL and TX	[20]
1916	Fatal case of possible DHF reported in TX	[20]
1922	Dengue epidemic, 500,000 to 600,000 cases in TX, 30,000 in Galveston, and 7,561 in LA	[20]
1923	1,376 dengue infections reported in LA	[20]
1924	1 dengue case in LA	[20]
1941–1944	Texas and gulf states involved in epidemic	[20]
1945	Last continental epidemic of dengue reported in LA	[20]
1980	1st indigenous dengue cases in USA since 1945 (Brownsville, TX), DENV-1 isolated	[21]
1981	DENV-4 cases reported, 1st isolation of DENV-4 in the USA	[20]
1982	1st reports of DENV-2 in the USA	[20]
1983	1st reports of DENV-3 in the USA	[20]
1986	DENV-1 reported in TX	[22]
1987	Autochthonous dengue reported in TX	[20]
1990	DENV-1, -2, and -3 isolated in the USA, reports of 102 dengue cases	[20]
1991	DENV-1 and DENV-3 isolated in the USA, 25 dengue cases reported	[20]
1994	91 cases of dengue, DENV-2 and -3 isolated in the USA	[20]
2005	First case of autochthonous DHF case reported in TX	[23]
2009–2011	Autochthonous dengue transmission in FL, DENV-1 isolated. DENV-1 isolated from a blood donor from Key West, FL in 2010	[24–26]
2012	4 DENV cases reported in FL, 2 of them in Miami-Dade	[26]
2013*	No indigenous dengue cases reported	[25]

Adapted from [20].

AL: Alabama, FL: Florida, GA: Georgia, LA: Louisiana, MO: Missouri, MS: Mississippi, PA: Pennsylvania, and TX: Texas.

*As of May 10, 2013.

**Table 2:** Imported and autochthonous cases of dengue reported in the state of Florida, USA, 2009–2013 (as of April 27, 2013).

Year	Number of imported cases	Countries visited* (number of cases)	Number of autochthonous cases	Florida counties (number of cases)
2009	36	BO (2), BR, CO (2), DO (3), GT (2), HT(10), HN (2), IN (3), MY, MX, NI, PA (3), PH, PR (3), SR	27	Monroe (27)
2010	133	BD, BR, KY, CO (8), CR (4), CU, DO (13), EC, SV, GH, GD (4), GT (2), HT (6), HN (6), JM (5), MQ (2), MV, MX, NI (13), PK, PH, PR (36), TH, TT, VE (16), VI (3), MY/AE/BD**, PA/VE**	65	Broward (1), Miami-Dade (1), and Monroe (63)
2011	61	AW, BS (14), BD (3), BR (3), CO, CR, CU (5), DO, GD, GY, HT (2), IN, JM (2), NI (2), PK, PA (2), PR (11), LC (2), TT (4), TC, VE, VN	7	Hillsborough (1), Martin (1), Miami-Dade (3), and Palm Beach (2)
2012	135	BR, CO, CU (29), DO (17), EC (4), SV (2), GH, GY (2), HT (17), HN, IN, JM (23), MX (2), NI (2), PA, PH (4), PT, PR (16), ZA, LK, VC, SR, TT (4), TC, VI	4	Miami-Dade (2), Seminole (1), and Osceola (1)
2013	27	AO, BB, BR, CO (2), DO (3), GT, HT, ID, JM (3), NG, PH, PR (8), MF	0	—

Total	392		103	

*AE: United Arab Emirates, AO: Angola, AW: Aruba, BB: Barbados, BD: Bangladesh, BO: Bolivia, BR: Brazil, BS: Bahamas, CO: Colombia, CR: Costa Rica, CU: Cuba, DO: Dominican Republic, EC: Ecuador, GD: Grenada, GH: Ghana, GT: Guatemala, GY: Guyana, HT: Haiti, HN: Honduras, ID: Indonesia, IN: India, JM: Jamaica, KY: Cayman Islands, LC: Saint Lucia, LK: Sri Lanka, MF: Saint Martin, MQ: Martinique, MY: Malaysia, MX: Mexico, MV: Maldives, NG: Nigeria, NI: Nicaragua, PA: Panama, PH: The Philippines, PK: Pakistan, PR: Puerto Rico, PT: Portugal, SR: Suriname, SV: El Salvador, TC: Turks and Caicos Islands, TH: Thailand, TT: Trinidad and Tobago, VC: St. Vincent and the Grenadines, VE: Venezuela, VI: US Virgin Islands, VN: Vietnam, and ZA: South Africa.

**Travel to more than one country.

Source: Florida State Department of Health [25, 26].

**Table 3:** Dengue activity in the state of Hawaii and the Territories of American Samoa, Guam, and Northern Mariana Islands, 1840s–2010.

Year(s)	Activity reported	References
Late 1840s	First large dengue epidemic recorded in HI, associated with *Aedes aegypti *	[34]
1903	Large dengue epidemic in HI, *≈*30,000 cases, associated with *Aedes aegypti *	[35]
1943-1944	DENV-1 epidemic in HI, *≈*1,500 cases	[34, 36]
1944	*Aedes aegypti* reported to be eradicated from GU. *Aedes albopictus *reported to be present in the island	[37]
<1950	Dengue cases were reported in AS and GU before 1950, no dengue epidemics reported in GU in recent times	[38]
1995	Possible dengue infection in German visitors to HI	[39]
1972	Dengue epidemic in AS (DENV-2)	[40]
1975	Dengue epidemic in AS (DENV-1)	[38, 40]
1995-1996	Dengue epidemic in AS (DENV-3)	[38]
1997	Reports of dengue cases in AS	[41]
1998	Dengue seropositive individuals reported in Saipan (MP) during 1998, DENV-2 implicated in epidemic activity	[42, 43]
2001	More than 1,600 dengue cases reported in AS (DENV-1), 3 deaths	[44]
	Dengue outbreak in the MP, >1,400 cases reported	[45]
2001-2002	Autochthonous transmission of dengue in HI, 122 confirmed cases, DENV-1 isolated, and *Aedes albopictus* was the implicated vector	[46]
2007	63 dengue cases confirmed in AS, 23 cases hospitalized	[47]
2008	Dengue activity reported in AS	[48]
2009	Outbreak of *≈*400 confirmed cases in AS	[48]
2010	Dengue cases reported in AS. Serosurvey conducted in 2010 revealed >95% of the tested individuals as seropositive for dengue	[8, 48]

AS: American Samoa, GU: Guam, HI: Hawaii, and MP: Northern Mariana Islands.

**Table 4:** Dengue activity in Puerto Rico and the USA Virgin Islands, 1915–2013.

Year(s)	Activity reported	References
1915	Dengue epidemic reported in PR	[20]
1924	1st recorded epidemic of dengue in the Caribbean-Gulf-Atlantic region begun in the VI	[20]
1941–1946	Dengue epidemic reported in PR	[20]
1963	Epidemic of *≈*27,000 cases (DENV-3) in PR	[55, 56]
1968-1969	DENV-2 (only) epidemic, 1st report of DENV-2 in PR, 16,665 cases	[57]
1970–1974	Sporadic DENV-2 cases reported in PR	[20, 57]
1975	DHF suspected among 3 serologically confirmed dengue cases, shock seen in 1 patient in PR - DHF described for the 1st time in the Western Hemisphere	[20, 57]
1977-1978	DENV-1 outbreak in PR, *≈*12,700 cases, 1st report of DENV-1 in PR, after DENV-2 and DENV-3 reports from earlier during that year	[58, 59]
1978	DENV-1 outbreaks in VI	[20]
1981–1983	DENV-1 and DENV-4 outbreaks in PR, 1st reports of DENV-4 in both PR and the Americas	[60, 61]
1985	2 DHF cases associated with DENV-4 in PR	[20, 62]
1986	Dengue epidemic in PR associated with DENV-4, 10,659 cases, 31 DHF cases, 3 deaths	[62]
1987	17 DHF cases in PR, 1 death	[20]
1988	8 DHF cases in PR	[20]
1989	DENV-1, -2, and -4 cases reported in PR, including 12 DHF cases, 5 deaths	[20]
	Dengue cases reported in the VI	[20]
1990	6 DHF cases in PR, 1 death	[20]
	Dengue cases reported, DENV-1, -2, and -4 involved in outbreaks, 1st report of DENV-4 in the VI	[20]
1991	14 DHF cases in PR, 1 death	[20]
1994	*≈*24,700 cases of dengue reported in PR	[63]
1998	>17,000 dengue cases reported in PR, 173 DHF cases, 9 deaths, all 4 serotypes isolated	[64]
1999	All 4 serotypes reported present in PR, 34 DHF cases (6 deaths), 4,993 dengue cases	[20]
2000	DENV-1, -2 and -3, isolated in PR, 24 DHF cases, 2,433 dengue cases	[20]
2005	Dengue reported in blood donors from PR, DENV-2 and DENV-3 isolated	[4]
	Dengue epidemic reported in St. Croix, VI	[65]
2007	Epidemic caused by DENV-3, -2, -1, and -4 (in order of frequency) in PR, more than 10,000 cases, 227 DHF cases, 40 deaths. Dengue reported in blood donors from PR	[66]
2010	Largest epidemic in PR history, DENV-1, -4, -2, and -3 isolated (in order of frequency), 26,766 cases reported, 448 DHF cases, 128 deaths. Dengue reported in blood donors from PR, DENV-1, DENV-4, and DENV-2 isolated	[67]
2012	Dengue epidemic in PR, 12,877 cases reported, DENV-1 and -4 isolated	[68]
	Dengue epidemic in the VI	[69]
2013*	Dengue epidemic in PR, >5,000 cases reported, DENV-1 and -4 isolated	[70]

Adapted from [20]. PR: Puerto Rico, VI: USA Virgin Islands.

*As of May 20, 2013.

## References

[1] Gubler DJ (1998). Dengue and dengue hemorrhagic fever. *Clinical Microbiology Reviews*.

[2] World Health Organization (1997). *Dengue Haemorrhagic Fever: Diagnosis, Treatment, Prevention and Control*.

[3] World Health Organization (2009). *Dengue: Guidelines for Diagnosis, Treatment, Prevention and Control*.

[4] Mohammed H, Linnen JM, Muhoz-Jorddn JL (2008). Dengue virus in blood donations, Puerto Rico, 2005. *Transfusion*.

[5] Tangnararatchakit K, Tirapanich W, Tapaneya-Olarn W (2012). Severe nonfebrile dengue infection in an adolescent after postoperative kidney transplantation: a case report. *Transplantation Proceedings*.

[6] Stramer SL, Linnen JM, Carrick JM (2012). Dengue viremia in blood donors identified by RNA and detection of dengue transfusion transmission during the 2007 dengue outbreak in Puerto Rico. *Transfusion*.

[7] Chen R, Vasilakis N (2011). Dengue—quo tu et quo vadis?. *Viruses*.

[8] Brady OJ, Gething PW, Bhatt S (2012). Refining the global spatial limits of dengue virus transmission by evidence-based consensus. *PLoS Neglected Tropical Diseases*.

[9] Guzman MG, Alvarez M, Halstead SB (2013). Secondary infection as a risk factor for dengue hemorrhagic fever/dengue shock syndrome: an historical perspective and role of antibody-dependent enhancement of infection. *Archives of Virology*.

[10] Bhatt S, Gething PW, Brady OJ (2013). The global distribution and burden of dengue. *Nature*.

[11] Gubler DJ (2002). Epidemic dengue/dengue hemorrhagic fever as a public health, social and economic problem in the 21st century. *Trends in Microbiology*.

[12] Añez G, Balza R, Valero N, Larreal Y (2006). Economic impact of dengue and dengue hemorrhagic fever in the State of Zulia, Venezuela, 1997–2003. *Revista Panamericana de Salud Publica*.

[13] Guzman MG, Halstead SB, Artsob H (2010). Dengue: a continuing global threat. *Nature Reviews Microbiology*.

[14] Whitehead SS, Blaney JE, Durbin AP, Murphy BR (2007). Prospects for a dengue virus vaccine. *Nature Reviews Microbiology*.

[15] Medlock JM, Hansford KM, Schaffner F (2012). A review of the invasive mosquitoes in Europe: ecology, public health risks, and control options. *Vector-Borne and Zoonotic Diseases*.

[16] Brown JE, Obas V, Morley V, Powell JR (2013). Phylogeography and spatio-temporal genetic variation of *Aedes aegypti* (Diptera: Culicidae) populations in the Florida Keys. *Journal of Medical Entomology*.

[17] Schaffner F, Medlock JM, Van Bortel W (2013). Public health significance of invasive mosquitoes in Europe. *Clinical Microbiology and Infection*.

[18] Mohammed HP, Ramos MM, Rivera A (2010). Travel-associated dengue infections in the United States, 1996 to 2005. *Journal of Travel Medicine*.

[19] Rush AB (1789). An account of the bilious remitting fever, as it appeared in Philadelphia in the summer and autumn of the year 1780. *Medical Enquiries and Observations*.

[20] Schneider J, Droll D A timeline for dengue in the Americas to December 31, 2000 and noted first occurrences. http://www.paho.org/English/HCP/HCT/dengue_timeline.xls.

[21] Centers for Disease Control and Prevention US (1980). Dengue—Texas. *Morbility and Mortality Weekly Reports*.

[22] Centers for Disease Control US, Prevention (1987). Imported and indigenous dengue fever—United States, 1986. *Morbility and Mortality Weekly Reports*.

[23] Malison MD, Waterman SH (1983). Dengue fever in the United States. A report of a cluster of imported cases and review of the clinical, epidemiologic, and public health aspects of the disease. *Journal of the American Medical Association*.

[24] Centers for Disease Control US, Prevention (2010). Locally acquired dengue—Key West, Florida, 2009-2010. *Morbility and Mortality Weekly Reports*.

[25] Florida State Department of Health http://www.doh.state.fl.us/Environment/medicine/arboviral/Weekly-Summary.html.

[26] Florida State Department of Health http://www.doh.state.fl.us/Environment/medicine/arboviral/weeklyreportarchive.html.

[27] Department of State US http://www.state.gov/documents/organization/155653.pdf.

[28] Knowlton K, Solomon G, Rotkin-Ellman M (2009). Mosquito-Borne dengue fever threat spreading in the Americas. *NRDC Issue Paper*.

[29] Benedict MQ, Levine RS, Hawley WA, Lounibos LP (2007). Spread of the tiger: global risk of invasion by the mosquito *Aedes albopictus*. *Vector-Borne and Zoonotic Diseases*.

[30] Hafkin B, Kaplan JE, Reed C (1982). Reintroduction of dengue fever into the continental United States. I. Dengue surveillance in Texas, 1980. *The American Journal of Tropical Medicine and Hygiene*.

[31] Centers for Disease Control US, Prevention (1996). Dengue fever at the U.S. Mexico border, 1995-1996. *Morbility and Mortality Weekly Reports*.

[32] Centers for Disease Control US, Prevention (2007). Dengue hemorrhagic fever—U.S. Mexico border, 2005. *Morbility and Mortality Weekly Reports*.

[33] Reiter P, Lathrop S, Bunning M (2003). Texas lifestyle limits transmission of dengue virus. *Emerging Infectious Diseases*.

[34] Gubler DJ, Gubler DJ, Kuno G (1997). Dengue and dengue hemorrhagic fever: its history and resurgence as a global public health problem. *Dengue and Dengue Hemorrhagic Fever*.

[35] Wilson GW (1904). Epidemic of dengue in the territory of Hawaii during 1903. *Public Health Reports*.

[36] Sabin AB (1952). Research on dengue during World War II. *The American journal of tropical medicine and hygiene*.

[37] Guillaumot L, Ofanoa R, Swillen L (2012). Distribution of *Aedes albopictus* (Diptera, Culicidae) in southwestern Pacific countries, with a first report from the Kingdom of Tonga. *Parasites & Vectors*.

[38] Kiedrzynski T, Souares Y, Stewart T (1998). Dengue in the Pacific: an updated history. *Pacific Health Dialog*.

[39] Imrie A, Zhao Z, Bennett SN, Kitsutani P, Laille M, Effler P (2006). Molecular epidemiology of dengue in the Pacific: introduction of two distinct strains of the dengue 2 type-1 virus into Hawaii. *Annals of Tropical Medicine and Parasitology*.

[40] Gratz NG, Knudsen AB *The Rise and Spread of Dengue, Dengue Haemorrhagic Fever and Its Vectors. An Historical Review (Up to 1995)*.

[41] PROMED PRO>Dengue—American Samoa.

[42] Singh N, Kiedrzynski T, Lepers C, Benyon EK (2005). Dengue in the Pacific—an update of the current situation. *Pacific Health Surveillance and Response*.

[43] Kiedrzynski T PRO/EDR>dengue—Saipan (04).

[44] Duncombe J, Lau C, Weinstein P (2013). Seroprevalence of dengue in American Samoa, 2010. *Emerging Infectious Diseases*.

[45] GIDEON

[46] Jelinek T, Dobler G, Nothdurft HD (1998). Evidence of dengue virus infection in a German couple returning from Hawaii. *Journal of Travel Medicine*.

[47] PROMED DENGUE/DHF UPDATE, 2007 (30).

[48] Arima Y, Matsui T (2011). Epidemiologic update on the dengue situation in the Western Pacific Region, 2010. *Western Pacific Surveillance and Response Journal*.

[49] Graham AS, Pruszynski CA, Hribar LJ (2011). Mosquito-associated dengue virus, Key West, Florida, USA, 2010. *Emerging Infectious Diseases*.

[50] Añez G, Heisey DA, Espina LM (2012). Phylogenetic analysis of dengue virus types 1 and 4 circulating in Puerto Rico and Key West, Florida, during 2010 epidemics. *The American Journal of Tropical Medicine and Hygiene*.

[51] Muñoz-Jordan JL, Santiago GA, Margolis H, Stark L (2013). Genetic relatedness of dengue viruses in Key West, Florida, USA, 2009-2010. *Emerging Infectious Diseases*.

[52] Usinger RI (1944). Entomological phases of the recent dengue epidemic in Honolulu. *Public Health Reports*.

[53] Gilbertson WE (1945). Sanitary aspects of the control of the 1943-44 epidemic of dengue fever in Honolulu. *American Journal of Public Health*.

[54] Effler PV, Pang L, Kitsutani P (2005). Dengue fever, Hawaii, 2001-2002. *Emerging Infectious Diseases*.

[55] Neff JM, Morris L, Gonzalez-alcover R, Coleman PH, Lyss SB, Negron H (1967). Dengue fever in a puerto rican community. *American Journal of Epidemiology*.

[56] Russell PK, Buescher EL, McCown JM, Ordõnez J (1966). Recovery of dengue viruses from patients during epidemics in Puerto Rico and East Pakistan. *The American Journal of Tropical Medicine and Hygiene*.

[57] Likosky WH, Calisher CH, Michelson AL (1973). An epidemiologic study of dengue type 2 in Puerto Rico, 1969. *American Journal of Epidemiology*.

[58] Morens DM, Rigau-Perez JG, Lopez-Correa RH (1986). Dengue in Puerto Rico, 1977: public health response to characterize and control an epidemic of multiple serotypes. *The American Journal of Tropical Medicine and Hygiene*.

[59] Gubler DJ, Thongcharoen P (1993). Dengue and dengue hemorrhagic in the Americas. *Dengue Hemorrhagic Fever*.

[60] Gubler DJ, Kuno G, Sather GE (1984). Mosquito cell cultures and specific monoclonal antibodies in surveillance for dengue viruses. *The American Journal of Tropical Medicine and Hygiene*.

[61] Waterman SH, Novak RJ, Sather GE (1985). Dengue transmission in two Puerto Rican communities in 1982. *The American Journal of Tropical Medicine and Hygiene*.

[62] Dietz V, Gubler DJ, Ortiz S (1996). The 1986 dengue and dengue hemorrhagic fever epidemic in Puerto Rico: epidemiologic and clinical observations. *Puerto Rico health sciences journal*.

[63] Rigau-Pérez JG, Vorndam AV, Clark GG (2001). The dengue and dengue hemorrhagic fever epidemic in Puerto Rico, 1994-1995. *The American Journal of Tropical Medicine and Hygiene*.

[64] Rigau-Pérez JG, Ayala-López A, García-Rivera EJ (2002). The reappearance of dengue-3 and a subsequent dengue-4 and dengue-1 epidemic in Puerto Rico in 1998. *The American Journal of Tropical Medicine and Hygiene*.

[65] Mohammed H, Ramos M, Armstrong J (2010). An outbreak of dengue fever in St. Croix (US Virgin Islands), 2005. *PLoS ONE*.

[66] Tomashek KM, Rivera A, Muñoz-Jordan JL (2009). Description of a large island-wide outbreak of dengue in Puerto Rico, 2007. *The American Journal of Tropical Medicine and Hygiene*.

[67] Sharp TM, Hunsperger E, Santiago GA (2013). Virus-specific differences in rates of disease during the 2010 dengue epidemic in Puerto Rico. *PLoS Neglected Tropical Diseases*.

[68] Departamento de Salud de Puerto Rico http://www.salud.gov.pr/Datos/VDengue/InfSemanales/Informe%20Semanal%202012/Informe%20Semana%20(Semana%2052).pdf.

[69] Centers for Disease Control US, Prevention (2012). Notes from the field: school reporting of a dengue outbreak—St. Croix, U.S. Virgin Islands. *Morbility and Mortality Weekly Reports*.

[70] Departamento de Salud de Puerto Rico http://www.salud.gov.pr/Datos/Dengue/Informes%20CDC%202013/Informe%20Dengue%20Semana%2013.pdf.

[71] PROMED PROMED-EDR: dengue—Samoa (2).

[72] Gubler D, Kuberski T, Rosen L Dengue in American Samoa.

[73] Burkot TR, Handzel T, Schmaedick MA, Tufa J, Roberts JM, Graves PM (2007). Productivity of natural and artificial containers for *Aedes polynesiensis* and *Aedes aegypti* in four American Samoan villages. *Medical and Veterinary Entomology*.

[74] McElroy KL, Santiago GA, Lennon NJ, Birren BW, Henn MR, Muñoz-Jordán JL (2011). Endurance, refuge, and reemergence of dengue virus type 2, Puerto Rico, 1986–2007. *Emerging Infectious Diseases*.

[75] Centers for Disease Control US, Prevention http://www.cdc.gov/dengue/resources/wklyrpt_eng/wklyrpt_eng.pdf.

[76] Linnen JM, Vinelli E, Sabino EC (2008). Dengue viremia in blood donors from Honduras, Brazil, and Australia. *Transfusion*.

[77] Kurolt IC, Betica-Radić L, Daković-Rode O (2013). Molecular characterization of dengue virus 1 from autochthonous dengue fever cases in Croatia. *Clinical Microbiology and Infection*.

[78] La Ruche G, Souarès Y, Armengaud A (2010). First two autochthonous dengue virus infections in metropolitan France, September 2010. *Euro Surveillance*.

[79] Morens DM, Fauci AS (2008). Dengue and hemorrhagic fever: a potential threat to public health in the United States. *Journal of the American Medical Association*.

[80] Adalja AA, Sell TK, Bouri N, Franco C (2012). Lessons learned during dengue outbreaks in the United States, 2001–2011. *Emerging Infectious Diseases*.

[81] Wilder-Smith A, Schwartz E (2005). Dengue in travelers. *The New England Journal of Medicine*.

[82] San Martín JL, Brathwaite O, Zambrano B (2010). The epidemiology of dengue in the Americas over the last three decades: a worrisome reality. *The American Journal of Tropical Medicine and Hygiene*.

[83] Gratz NG (2004). Critical review of the vector status of *Aedes albopictus*. *Medical and Veterinary Entomology*.

[84] Goh KT, Ng SK, Kumarapathy S (1985). Disease-bearing insects brought in by international aircraft into Singapore. *Southeast Asian Journal of Tropical Medicine and Public Health*.

[85] Service MW (1997). Mosquito (Diptera: Culicidae) dispersal—the long and short of it. *Journal of Medical Entomology*.

[86] Eritja R, Ramos HD, Aranda C (2000). Aircraft-mediated mosquito transport: new direct evidence. *Journal of the American Mosquito Control Association*.

[87] Tatem AJ, Hay SI, Rogers DJ (2006). Global traffic and disease vector dispersal. *Proceedings of the National Academy of Sciences of the United States of America*.

[88] Iturbe-Ormaetxe I, Walker T, O’Neill SL (2011). Wolbachia and the biological control of mosquito-borne disease. *EMBO Reports*.

